# Intensive Care Unit-Acquired Weakness after Liver Transplantation: Analysis of Seven Cases and a Literature Review

**DOI:** 10.3390/jcm12247529

**Published:** 2023-12-06

**Authors:** Rita Gaspari, Giorgia Spinazzola, Paola Aceto, Alfonso Wolfango Avolio, Manuel Delli Compagni, Stefania Postorino, Teresa Michi, Daniele Cosimo Fachechi, Anna Modoni, Massimo Antonelli

**Affiliations:** 1 Department of Emergency, Anesthesiologic and Reanimation Sciences, Fondazione Policlinico Universitario Agostino Gemelli IRCCS, 00168 Rome, Italy; rita.gaspari@unicatt.it (R.G.); giorgia.spinazzola@policlinicogemelli.it (G.S.); manuel.dellicompagni01@icatt.it (M.D.C.); stefania.postorino@policlinicogemelli.it (S.P.); teresa.michi@policlinicogemelli.it (T.M.); danielecosimo.fachechi@guest.policlinicogemelli.it (D.C.F.); massimo.antonelli@unicatt.it (M.A.); 2 Department of Basic Biotechnological Science, Intensive and Peri-Operative Clinics, Catholic University of the Sacred Heart, 00168 Rome, Italy; 3 Department of Translational Medicine and Surgery, Catholic University of the Sacred Heart, 00168 Rome, Italy; alfonso.avolio@unicatt.it; 4 General Surgery and Liver Transplantation, Fondazione Policlinico Universitario Agostino Gemelli IRCCS, 00168 Rome, Italy; 5 Department of Geriatric, Neurologic, Orthopedics and Head-Neck Science, Area of Neuroscience, Institute of Neurology, Fondazione Policlinico Universitario Agostino Gemelli IRCCS, 00168 Rome, Italy; anna.modoni@policlinicogemelli.it

**Keywords:** critical illness polyneuropathy, critical illness myopathy, neurophysiological studies, liver transplant, early allograft dysfunction, red blood cell transfusions

## Abstract

Intensive Care Unit (ICU)-Acquired Weakness (ICU-AW) is a generalized muscle weakness that is clinically detected in critical patients and has no plausible etiology other than critical illness. ICU-AW is uncommon in patients undergoing orthotopic liver transplantation (OLT). Our report sheds light on the highest number of ICU-AW cases observed in a single center on OLT patients with early allograft dysfunction. Out of 282 patients who underwent OLT from January 2015 to June 2023, 7 (2.5%) developed generalized muscle weakness in the ICU and underwent neurophysiological investigations. The neurologic examination showed preserved extraocular, flaccid quadriplegia with the absence of deep tendon reflexes in all patients. Neurophysiological studies, including electromyography and nerve conduction studies, showed abnormalities with fibrillation potentials and the rapid recruitment of small polyphasic motor units in the examined muscles, as well as a reduced amplitude of the compound muscle action potential and sensory nerve action potential, with an absence of demyelinating features. Pre-transplant clinical status was critical in all patients. During ICU stay, early allograft dysfunction, acute kidney injury, prolonged mechanical ventilation, sepsis, hyperglycemia, and high blood transfusions were observed in all patients. Two patients were retransplanted. Five patients were alive at 90 days; two patients died. In non-cooperative OLT patients, neurophysiological investigations are essential for the diagnosis of ICU-AW. In this setting, the high number of red blood cell transfusions is a potential risk factor for ICU-AW.

## 1. Background

Generalized clinical weakness, occurring during Intensive Care Unit (ICU) hospitalization and for which no cause other than critical illness can be identified, is indicated as “ICU-acquired weakness” (ICU-AW) [1]. The incidence of ICU-AW reported in critical illness ranges from 25 to 50%, reaching 67% in patients with prolonged mechanical ventilation (longer than 7 days) [2]. This variability is due to the heterogeneity of the studied population, the diagnostic technique used, and the timing of the assessment. ICU-AW can be evoked by critical illness polyneuropathy (CIP) [3], critical illness myopathy (CIM), or both [4].

The clinical presentation varies in severity from mild muscle weakness to complete paralysis. It is usually represented by symmetrical flaccid paralysis prevalent in the lower limbs and respiratory muscle weakness. Cranial nerves, including oculomotor nerves, with bulbar innervation are generally spared.

ICU-AW should be suspected when symptoms cannot be attributed to primary neuromuscular disorders (<0.5% of all ICU admissions), i.e., myasthenia gravis, amyotrophic lateral sclerosis or multiple sclerosis, and Guillain-Barré Syndrome [5].

ICU-AW is associated with delayed weaning from mechanical ventilation, prolonged ICU stay, higher hospitalization costs, and a high overall mortality rate. The main risk factors include high severity of the illness at ICU admission, sepsis, multiple organ failure, neuromuscular block for 3–5 days, large cumulative doses of corticosteroids, aminoglycoside, prolonged bed rest or immobility, and hyperglycemia [1,6]. Furthermore, females and the elderly seem to be the most affected by this disorder [7,8].

Prevention can be attempted by managing modifiable risk factors such as hyperglycemia induced by both severe stress and parenteral nutrition [9,10]. Moreover, several drugs frequently used in critically ill patients have been associated with the risk of ICU-AW, including vasoactive medications, especially in cases of high doses and long-lasting administrations [11], corticosteroids [12], and some antibiotics, including aminoglycosides and vancomycin [9]. The contribution of neuromuscular blocking agents (NMBAs) to the occurrence of ICU-AW is unclear [9]. Two studies showed contrasting results regarding the impact of a 48 h infusion of cisatracurium on the risk of ICU-AW [13,14]. However, NMBAs may promote muscle weakness in cases of infusion duration >48 h or the concomitant administration of corticosteroids [15]. Sedation-induced immobility can be the main indirect negative effect of hypnotic drugs in ICU-AW patients [16].

ICU-AW is characterized by functional and structural alterations of both nerves and muscles. In CIP, the pathological finding is axonal degeneration [17], while in CIM the histological study of muscle biopsy specimens documents the loss of myosin, the presence of acute necrotizing myopathy, and the cachectic condition [1,18]. The pathophysiological mechanisms underlying the occurrence of ICU-AW are not completely understood and include alterations in microcirculation, metabolic disorders, the direct toxic effects of ICU therapies, abnormalities in electrical transmission, and “bioenergetic” problems [19]. Pathophysiological mechanisms contribute independently, simultaneously, or synergistically to the pathogenesis of CIP and CIM.

A clinical quantification of muscle strength should be performed to diagnose ICU-AW, but it requires the patient’s cooperation. Electrophysiological examination is focused on electromyography and nerve conduction studies. Both tests show abnormalities with fibrillation potentials and the rapid recruitment of small polyphasic motor units in the examined muscles. Furthermore, a reduced amplitude of the compound muscle action potential and sensory nerve action potential, with the absence of demyelinating features, can be observed [20].

Electrophysiological examination, which can also be applied to unconscious/uncooperative patients, is helpful for establishing both diagnosis and prognosis [21]. The loss of muscle mass exceeding 10% over the first week in the ICU foresees functional impairment [22,23]. In the absence of electrophysiological abnormalities, severe disuse muscle atrophy has been proposed as a separate kind of weakness [24,25].

ICU-AW has rarely been reported after orthotopic liver transplantation (OLT) [26,27,28,29,30,31]. However, there are four brief reports in the literature, each describing 1–4 cases for a total number of 9 cases (Table 1). Here we illustrate the largest experience in ICU-AW after OLT in order to identify the risk factors linked to the disorder.

## 2. Analysis of Seven Cases

**Figure 1:**
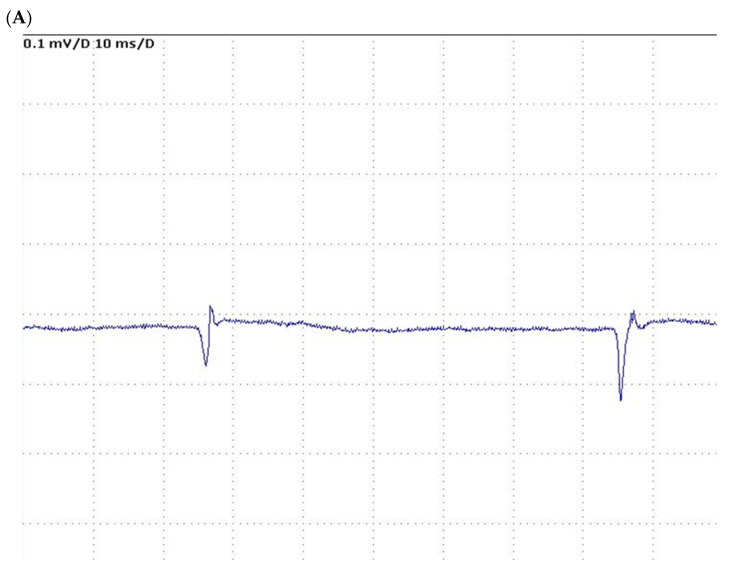

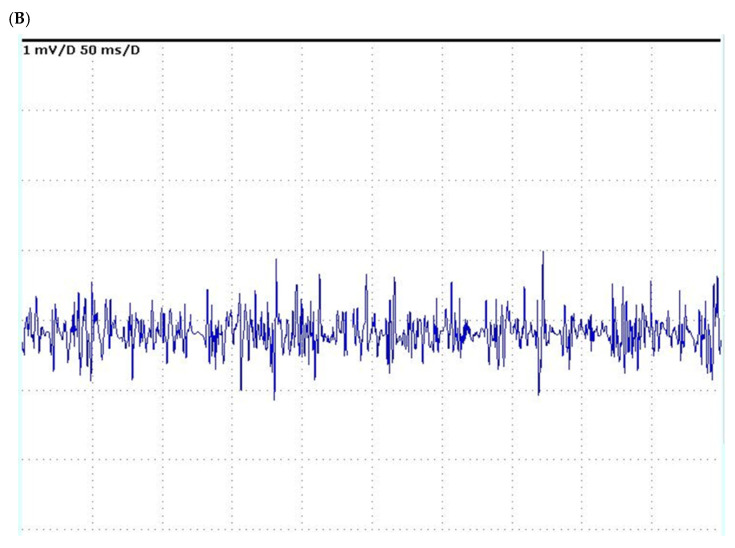
(**A**): Needle EMG, spontaneous activity with sporadic fibrillation potentials. (Amplitude: 0.1 mV/Division; duration 10 ms/Division). (**B**): Needle EMG, early recruitment with low amplitude, full interference pattern at a less-than-maximal effort of contraction. (Amplitude: 1 mV/Division; duration 50 ms/Division).

Between January 2015 and June 2023, 7 (2.5%) out of 282 patients admitted to the ICU, after OLT from deceased donors, developed muscle weakness. In these patients, an electrophysiological study was performed in the ICU using standardized techniques according to Kimura’s principles [32] with a portable Keypoint electromyography machine. Motor and orthodromic sensory nerve conductions were recorded using surface electrodes after surface stimulation; amplitudes were measured from peak to peak for motor responses and baseline to negative peak for sensory responses. Motor nerve conduction was recorded from the median and ulnar nerves. Sensory nerve conduction was recorded from the sural, radial, and ulnar nerves. Nerve conduction studies (NCS) were performed bilaterally, including the motor conduction studies of the right median and ulnar nerves as well as sensory conduction studies of the right median and ulnar nerves and of the right and left sural nerves. Surface electrodes were used for nerve stimulation and recording. In addition, needle electromyography (EMG) was performed with concentric electrodes in the deltoid, biceps, and rectus femoris muscles. Skin temperature was maintained above 35 °C during all recordings. EMG demonstrated myopathic abnormalities with fibrillation potentials and the rapid recruitment of small polyphasic motor units in the examined muscles of all subjects. NCS revealed a decrease in the amplitudes of compound motor action potentials and sensory nerve action potentials without demyelinating characteristics. Clinical neurologic examination showed preserved extraocular, mimic, and tongue muscles, and flaccid quadriplegia with the absence of deep tendon reflexes. The EMG patterns of the deltoid muscle in Case 7 are shown in Figure 1.

General clinical and surgical characteristics are summarized in Table 2. Relevant data are described as mean ± standard deviation. Generalized muscle weakness appeared 10.0 ± 2.5 days after OLT. No patient showed any clinical signs of muscle weakness before surgery. The pre-transplant clinical status was critical in all patients (six of them had been admitted to the ICU before the transplant). The patients’ mean age was 50 ± 10.7 years, while their body mass index was 27.5 ± 4.6, their Model for End-stage Liver Disease (MELD) was 36 ± 4.5, and their Simplified Acute Physiology Score II was 53.3 ± 15.0. Intraoperative transfusions consisted of red blood cell (RBC) 16.1 ± 10.7 units, fresh-frozen plasma (FFP) 11.1 ± 7.9 units, and platelet 3.3 ± 3.0 units. All patients received tacrolimus with mycophenolate mofetil and corticosteroids as first-line immunosuppressive treatment. Two patients showed high Early Allograft failure Simplified Estimation (EASE) score values and experienced early allograft failure [33]. Both were retransplanted on the third postoperative day. In contrast, the remaining five patients experienced varying degrees of primary dysfunction [34], although they showed recovery of graft function during their stay in the ICU. Allograft rejection was diagnosed in one patient. Continuous renal replacement therapy for acute kidney injury (AKI) was carried out in all patients. The duration of mechanical ventilation was 190.7 ± 81.4 h due to difficulty in weaning. Three patients were tracheostomized. No one received neuromuscular blockers for more than 24 h, only sedatives. Systematic bacteriological studies are shown in Table 1.

All patients were hyperglycemic and were treated with insulin infusion, although only one patient suffered from non-insulin-dependent diabetes mellitus prior to transplantation. All patients received a high number of transfusions (RBCs ≥ 15 units, FFP > 6 units, platelets > 3 units) during and/or very close to transplantation. The postoperative serum creatine phosphate kinase (CPK) level reached 2000 IU/L in one patient. CPK levels in the remaining patients were only slightly elevated, but then returned to normal levels.

ICU length of stay was 37.0 ± 22.9 days. Two patients died during ICU stay (on the 33rd and 40th postoperative day, respectively). A follow-up examination at 90 days showed the progressive clinical recovery of muscle strength in five patients. The management of the patient consisted of early and intensive physiotherapy (stepwise mobilization with a passive range of motion in bed), blood glucose control, early enteral nutrition [35], and infectious surveillance in addition to daily administration of L-acetyl cysteine (500 mg i.v. twice a day), thiamine 100 mg i.m., and daily supplementation i.v. of vitamin complex (A, B, C, and D).

All patients provided written informed consent for their data to be collected and analyzed for scientific purposes before OLT.

## 3. Literature Review and Discussion

This is the largest experience of ICU-AW after liver transplantation with an incidence of 2.5%. In this setting, the diagnosis of ICU-AW can be complex due to overlapping symptoms that influence the postoperative course.

In sedated or comatose patients, the neurophysiological study is the essential method to formulate the final or differential diagnosis. It usually includes nerve conduction studies, electromyography, and more complex electrophysiological methods such as direct muscle stimulation [4]. Muscle biopsy is rarely used to diagnose ICU-AW due to patient rejection, pain, or the presence of coagulopathy. The use of serum creatine phosphate kinase level as a biomarker of ICU-AW is not useful for diagnosis due to non-specificity [36]. Point-of-care neuromuscular ultrasound is emerging as a potential non-invasive marker of ICU-AW [37]. However, further studies demonstrating an association between ultrasound findings and clinical outcomes are required.

Neuromuscular dysfunctions complicating the postoperative course of liver transplantation have rarely been reported in the literature. So far, mononeuropathy or myopathy, rather than polyneuropathy, have been identified as the cause of ICU-AW after surgery. We should distinguish cases with polyneuropathy and/or myopathy as those reported in the present series from cases with mononeuropathy. While polyneuropathy is characterized by a “systemic condition”, mononeuropathy mainly occurs after iatrogenic trauma to a nerve or plexus.

Yet, in liver transplanted cases, the largest case series has been reported by Mirò et al. The authors documented myosin-loss myopathy in 4 of 281 patients with acute quadriplegic weakness, but no electrodiagnostic tests were performed [26]. Using electrophysiological studies on 520 patients, Wijdicks et al. found mononeuropathy and acute necrotic myopathy in 9 and 5 patients, respectively, whilst rhabdomyolysis was found using muscle biopsy in only 1 patient [27]. Rezaiguia-Delclaux et al. found sensorimotor axonal polyneuropathy at electrophysiological testing in 3 of 30 subjects with acute quadriplegia after liver transplantation [28]. More recently, two additional cases of ICU-AW were reported in patients undergoing liver transplantation from a living donor [29,30]. In both cases, the patients developed acute respiratory distress syndrome requiring prolonged mechanical ventilation. Electrophysiological studies revealed myopathy in one case [29] and axonal degeneration of sensory and motor fibers in the other [30]. Notably, Campellone et al., in 7 subjects out of 77, found only mononeuropathy on electrophysiological examination [31]. Intra-operative iatrogenic damage was identified in all cases.

In our study, we identified the same risk factors for neuromuscular dysfunction reported by the aforementioned authors, such as poor allograft function and acute kidney injury (Table 2). The enhanced muscle protein catabolism, common in these conditions, might be a possible contributing factor for developing ICU-AW. However, given that most of our patients, five out of seven, showed recovered graft function during ICU stay, the association between ICU-AW and graft dysfunction could be only concomitant, i.e., associated with the extended ICU stay, but not causal. It is reasonable that the postoperative course of patients with severe graft dysfunction—especially those with trajectories to early graft failure—is characterized by a prolonged ICU stay until graft loss and retransplant or death occur.

All of our patients showed decompensated clinical status before transplantation as evidenced by the MELD score. A high MELD score usually predicts longer postoperative ICU stay, prolonged mechanical ventilation, and/or higher risk of infection [38]. We believe the diagnosis of ICU-AW should be obtained by means of the electrophysiological study since the muscle biopsy in this setting implies an unsustainable bleeding risk due to coagulopathy and thrombocytopenia.

Unfortunately, the donor–recipient matching was unfavorable in five out of seven cases due to the impossibility of waiting for a younger donor because of the deteriorated conditions of the recipients [39]. Contrary to the cases reported in the literature, only one case in our series was transplanted for HCV interferon-treated cirrhosis [40]. However, none of our patients received extensive administration of neuromuscular blocking agents for prolonged mechanical ventilation, only sedatives. Regarding tacrolimus, although we cannot exclude its toxic effect, we observed an improvement in ICU-AW despite its continuation at therapeutic blood levels. Only one of our patients received a higher dose of corticosteroids for acute rejection. Concerning the treatment of infections, we did not use the aminoglycosides mentioned among the possible risk factors of ICU-AW. We kept serum glucose levels in the normal range through insulin infusion for all infections. We observed an increase in serum creatine phosphate kinase levels in only one patient, while in the remaining six patients the slight increase in creatine phosphate kinase, usually appreciable in the early postoperative period, did not suggest necrotizing myopathy.

Finally, as already reported [26], we noticed that all of our patients received large blood transfusions during OLT and in the perioperative period (Table 1 and Table 2). Although the transfusion threshold has decreased in recent decades, liver transplantation—due to its technical complexity or the critical illness of the patient—may require massive transfusions [41,42]. Unfortunately, we do not know the pathogenetic mechanism by which a large blood transfusion can cause neuromuscular damage and contribute to the appearance of ICU-AW. Mirò et al. [26] supposed that the high transfusion load could have played a role in their patients due to sudden and repeated changes in extracellular electrolyte levels, especially ionized calcium (perhaps mediated by citrate) and pH during blood replacement therapy. All these modifications may increase muscle susceptibility to damage through concomitant myopathic adverse factors, including corticosteroids, neuromuscular blocking drugs, hyperglycemia, and surgical injury. Impaired microcirculation due to microthrombi and microvascular alterations in the endothelium, systemic inflammation, direct toxic effects on mitochondria, and increased serum levels of cortisol and catecholamines caused by severe and prolonged post-hemorrhagic hypotensive episodes can play a role in the pathophysiologic process. Furthermore, large blood transfusions can cause lung injury (edema, transfusion-related acute lung injury), prolong mechanical ventilation, as observed in our patients, and contribute to protracted immobilization. It is likely that the association between ICU-AW and a high RBC transfusion load during surgery and the early postoperative period only reflects the severity of critical end-stage liver disease. The need for transfusion is mainly related to a high MELD score [43] and to other factors that increase the risk of bleeding, such as pre-transplant thrombocytopenia, portal hypertension, portal vein thrombosis, prior upper abdominal surgery, and previous transplant [43,44]. High transfusion load is strictly associated with the perioperative dysfunction of the fibrinolytic system that plays a role in affecting the outcomes of liver transplant recipients, leading to a prolonged length of stay in the ICU [45]. The consequent systemic microvascular fibrin deposition, which has been advocated as an underlying mechanism of early allograft dysfunction [46], could have a potential role in the physiopathology of ICU-AW. Although clinical data are currently still lacking, a study on a graft ischemia/reperfusion injury model in rats revealed that increased fibrin deposition in muscle tissue could be responsible for its damage [47]. This interesting finding still needs to be further explored. Interestingly, two out of seven patients with ICU-AW were obese (BMI > 30 kg/m^2^), while three were overweight (BMI > 25 kg/m^2^). However, recent evidence shows that obesity attenuates sepsis-induced muscle wasting and weakness in mice [48]. The more pronounced lipolysis could be responsible for muscle protection observed in overweight/obese septic mice [48]. However, sarcopenic obesity is frequent among liver transplant recipients; this could be the reason why their muscles were not protected against the catabolic state driven by the stress response during the early phase of critical illness. Sarcopenic obesity, referred to as the combination of obesity with low skeletal muscle mass and function, might have played a key role in the acquired muscle weakness. However, this cannot be confirmed in our cases, as skeletal muscle mass index (SMI) was not measured before liver transplant, as was measured in a previously published case report of a non-obese sarcopenic patient with post-OLT ICU-AW [28]. In the latter case report, the patient’s SMI—calculated using bioimpedance—constantly increased during physical rehabilitation, thus confirming its effectiveness.

The current guidelines recommend bedside manual testing of muscle strength in awake and cooperative patients, whilst severity is scored using the Medical Research Council’s (MRC’s) variant sum-score [49]. The MRC’s sum-score ranges between 0 and 60, and a score of <48 suggests ICU-AW. This scale has some limitations: it identifies muscle weakness but does not distinguish between CIP and CIM. Moreover, it is subject to significant inter-observer variation and requires full patient collaboration. Therefore, the routine use of the MRC’s sum-score in the ICU is rather limited because patients are often sedated, comatose, or severely disoriented. Even the assessment of force in the manual handpiece with subjective evaluation or using a dynamometer, although reliable, shows the same limitations as the MRC’s sum-score test in the ICU [50]. In the present series, the MRC’s sum-score [51] was not carried out because the patients were comatose or severely encephalopathic.

Regarding the clinical management of ICU-AW, although no specific action has been demonstrated to improve the prognosis, the minimization of risk factors such as the early treatment of sepsis, tight blood glucose control with the aim of normoglycemia according to the NICESUGAR guidelines, the limited use of muscle relaxants and corticosteroids, vitamin administration, nutritional support, and early mobilization associated with an intense physiotherapy program have proven beneficial [52].

Recovery usually occurs within weeks to months, although it may be incomplete, with weakness persisting for up to 2 years after ICU discharge. Prognosis appears impaired when the cause of ICU-AW involves critical illness polyneuropathy, whereas isolated critical illness myopathy may have a better prognosis [53].

## 4. Conclusions

Patients undergoing liver transplantation, especially the most critically ill ones, may present several risk factors for the development of ICU-AW in the postoperative course. So far, only one study has reported large blood transfusions as a possible risk factor for this complication.

Although our data confirm this finding, we hypothesize a multifactorial genesis. The prognosis of ICU-AW after liver transplantation remains favorable, although a long recovery should be expected. The early identification of other complications is of paramount importance.

The definition of the best immunosuppressive regimen and the validation of the role of blood transfusions on the occurrence of ICU-AW in critically ill liver transplanted patients should be explored in future studies.

## Figures and Tables

**Table 1:** Overview of articles reporting Intensive Care Unit-Acquired Weakness in liver transplanted patients.

Author, Year	Age, Years	Gender, M/F	Transplant Donor	Primary Disease	Child-Pugh/ MELD	Graft Function	^ RBC Units; * RBC Units	AKI	Re-Transplant	Discharge
Mirò O, 1999 [26] Incidence 4/281 (1.4%)										
Case 1	51	M	DD	ALC	CP 6 (A)	Poor	9; 11	Yes	Yes	89 POD
Case 2	57	M	DD	HCV	CP 8 (B)	PNF	21; 46	Yes	Yes	229 POD
Case 3	41	F	DD	HCV	CP 13 (C)	Good	68; 9	Yes	Yes	48 POD
Case 4	59	M	DD	HCV	CP 5 (A)	Poor	4; 26	Yes	Yes	46 POD
Watanabe J, 2016 [30]	43	M	LD	HCV	MELD 20	Poor	NS	Yes	Yes	150 POD
Jang MH, 2018 [29]	47	M	LD	HBV	MELD 25	NS	NS	HS	No	91 POD
Rezaiguia-Delclaux S, 2022 [28] Incidence 3/30 (10%)										
Case 1	59	M	DD	ALC	CP 10 (C)	PNF	NS	Yes	Yes	Discharged ^§^
Case 2	42	M	DD	ALC	CP 10 (C)	PNF	NS	Yes	Yes	Death (157th day)
Case 3	60	M	DD	ALC	CP 11 (C)	PVT	NS	No	RS	Discharged ^§^

Abbreviations. DD: deceased donor; LD: living donor; HCV/HBV: hepatitis B virus/hepatitis C virus; ALC: alcohol; CP: Child–Pugh; MELD: Model for End-stage Liver Disease; PNF: primary non-function; PVT: Portal Vein Thrombosis; RBCs: red blood cells; AKI: acute kidney injury; RS: repair stent; NS: not stated; POD: postoperative day. ^: intraoperative transfusion; *: perioperative transfusion; ^§^ discharge day not reported.

**Table 2:** Characteristics of patients affected by Intensive Care Unit-Acquired Weakness after liver transplantation.

Parameters	Case 1	Case 2	Case 3	Case 4	Case 5	Case 6	Case 7
Age, years	64	56	58	38	35	46	53
Gender, M/F	M	M	F	F	M	M	F
BMI, Kg/m^2^	26.6	22.2	34.2	33.5	26.1	25.2	24.6
Alcoholic cirrhosis	No	Yes	No	Yes	No	No	No
HBV/HCV Cirrhosis	No	Yes	No	No	No	No	No
Other causes of LD	Cryptogenic	-	Polycystosis	-	Cryptogenic	Trauma	AID
Hepatocarcinoma	No	Yes	No	No	No	No	No
Diabetes mellitus	No	No	Yes	No	No	No	No
MELD score	30	38	40	40	40	31	33
D-MELD score	810	912	3105	3160	2420	2139	2234
^ RBCs, units	36	18	19	11	19	6	4
^ FFP, units	23	14	8	5	20	5	3
^ Platelets, units	9	5	3	2	3	0	1
SAPS II score	46	76	49	60	41	67	34
EASE score	−0.9	+1.9	+1.7	−1.3	−1.9	−1.7	−2.1
P.o. Hyperglycemia	Yes	Yes	Yes	Yes	Yes	Yes	Yes
Tracheostomy	Yes	No	No	No	No	Yes	Yes
Norepinephrine	Yes	Yes	Yes	Yes	Yes	Yes	Yes
Serum CPK, UI/L	74	113	239	15	2000	80	120
Pre-OLT ICU stay, days	0	4	5	9	1	13	3
Pre-OLT CRRT	No	Yes	No	Yes	Yes	Yes	No
Post-OLT CRRT	Yes	Yes	Yes	Yes	Yes	Yes	Yes
EAF	No	Yes	Yes	No	No	No	No
* RBCs ≥ 15, units	Yes	Yes	Yes	Yes	Yes	Yes	Yes
Graft Rejection	No	No	No	No	No	No	Yes
Infection site	Blood	Blood/Bal	Blood	Urine	Blood/Bal	Blood/ascites	Blood
Duration of MV, hours	168	75	144	336	192	180	240
ICU-AW post-OLT, days	14	8	8	8	10	13	9
ICU-AW recovery	Yes	Yes	Yes	Yes	Yes	Yes	No
ICU-LOS, days	79	14	20	20	33	53	40
Hospital LOS, days	187	53	68	85	33	177	40
90 days outcome	Alive	Alive	Alive	Alive	Dead	Alive	Dead

Abbreviations. BMI: body mass index; HBV/HCV: hepatitis B virus/hepatitis C virus; LD: liver disease; AID: autoimmune diseases; MELD: Model for End-stage Liver Disease; D-MELD: donor age for MELD; RBCs: red blood cells; FFP: fresh-frozen plasma; SAPS: Simplified Acute Physiology Score; EASE: Early Allograft failure Simplified Estimation score; EAF: early allograft failure; CPK: creatine phosphate kinase; ICU: Intensive Care Unit; OLT: orthotopic liver transplantation; CRRT: continuous renal replacement therapy; MV: mechanical ventilation; LOS: length of stay. ^: intraoperative transfusion; *: perioperative transfusion.

## Data Availability

The presented data are available in the patients’ medical records.

## References

[1] Latronico N., Bolton C.F. (2011). Critical illness polyneuropathy and myopathy: A major cause of muscle weakness and paralysis. Lancet Neurol..

[2] Ali N.A., O’Brien J.M., Hoffmann S.P., Phillips G., Garland A., Finley J.C., Almoosa K., Hejal R., Wolf K.M., Lemeshow S. (2008). Acquired weakness, handgrip strength, and mortality in critically ill patients. Am. J. Respir. Crit. Care Med..

[3] Bolton C.F., Gilbert J.J., Hahn A.F., Sibbald W.J. (1984). Polyneuropathy in critically ill patients. J. Neurol. Neurosurg. Psychiatry.

[4] Stevens R.D., Marshall S.A., Cornblath D.R., Hoke A., Needham D.M., de Jonghe B., Ali N.A., Sharshar T. (2009). A framework for diagnosing and classifying intensive care unit-acquired weakness. Crit. Care Med..

[5] Damian M.S., Wijdicks E.F.M. (2019). The clinical management of neuromuscular disorders in intensive care. Neuromuscul. Disord..

[6] Zhou C., Wu L., Ni F., Ji W., Wu J., Zhang H. (2014). Critical illness polyneuropathy and myopathy: A systematic review. Neural. Regen. Res..

[7] De Jonghe B., Sharshar T., Lefaucheur J.P., Authier F.J., Durand-Zaleski I., Boussarsar M., Cerf C., Renaud E., Mesrati F., Carlet J. (2002). Paresis acquired in the intensive care unit: A prospective multi-center study. JAMA.

[8] Patel B.K., Pohlman A.S., Hall J.B., Kress J.P. (2014). Impact of early mobilization on glycemic control and ICU-acquired weakness in critically ill patients who are mechanically ventilated. Chest.

[9] Yang T., Li Z., Jiang L., Wang Y., Xi X. (2018). Risk factors for intensive care unit-acquired weakness: A systematic review and meta-analysis. Acta Neurol. Scand..

[10] Hermans G., Casaer M.P., Clerckx B., Güiza F., Vanhullebusch T., Derde S., Meersseman P., Derese I., Mesotten D., Wouters P.J. (2013). Effect of tolerating macronutrient deficit on the development of intensive-care unit acquired weakness: A subanalysis of the EPaNIC trial. Lancet Respir. Med..

[11] Wolfe K.S., Patel B.K., MacKenzie E.L., Giovanni S.P., Pohlman A.S., Churpek M.M., Hall J.B., Kress J.P. (2018). Impact of vasoactive medications on ICU-acquired weakness in mechanically ventilated patients. Chest.

[12] Yang T., Li Z., Jiang L., Xi X. (2018). Corticosteroid use and intensive care unit-acquired weakness: A systematic review and meta-analysis. Crit. Care.

[13] Papazian L., Forel J.M., Gacouin A., Penot-Ragon C., Perrin G., Loundou A., Jaber S., Arnal J.M., Perez D., Seghboyan J.M. (2010). ACURASYS Study Investigators Neuromuscular blockers in early acute respiratory distress syndrome. N. Engl. J. Med..

[14] Moss M., Huang D.T., Brower R.G., Ferguson N.D., Ginde A.A., Gong M.N., Grissom C.K., Gundel S., Hayden D., Hite R.D. (2019). Early neuromuscular blockade in the acute respiratory distress syndrome. N. Engl. J. Med..

[15] Bourenne J., Hraiech S., Roch A., Gainnier M., Papazian L., Forel J.M. (2017). Sedation and neuromuscular blocking agents in acute respiratory distress syndrome. Ann. Transl. Med..

[16] Foster J. (2016). Complications of sedation in critical illness: An update. Crit. Care Nurs. Clin. N. Am..

[17] Latronico N., Fenzi F., Recupero D., Guarneri B., Tomelleri G., Tonin P., De Maria G., Antonini L., Rizzuto N., Candiani A. (1996). Critical illness myopathy and neuropathy. Lancet.

[18] Stibler H., Edström L., Ahlbeck K., Remahl S., Ansved T. (2003). Electrophoretic determination of the myosin/actin ratio in the diagnosis of critical illness myopathy. Intensive Care Med..

[19] De Jounghe B., Lacherade J.C., Sharshar T., Outin H. (2009). Intensive care unit-acquired weakness: Risk factors and prevention. Crit. Care Med..

[20] Appleton R.T., Kinsella J., Quasim T. (2015). The incidence of intensive care unit-acquired weakness syndromes: A systematic review. J. Intensive Care Soc..

[21] Vanhorebeek I., Latronico N., Van den Berghe G. (2020). ICU-acquired weakness. Intensive Care Med..

[22] Puthucheary Z.A., Rawal J., McPhail M., Connolly B., Ratnayake G., Chan P., Hopkinson N.S., Phadke R., Dew T., Sidhu P.S. (2013). Acute skeletal muscle wasting in critical illness. JAMA.

[23] Derde S., Hermans G., Derese I., Güiza F., Hedström Y., Wouters P.J., Bruyninckx F., D’Hoore A., Larsson L., Van den Berghe G. (2012). Muscle atrophy and preferential loss of myosin in prolonged critically ill patients. Crit. Care Med..

[24] Latronico N., Rasulo F.A., Eikermann M., Piva S. (2023). Illness Weakness, Polyneuropathy and Myopathy: Diagnosis, treatment, and long-term outcomes. Crit. Care.

[25] Latronico N., Herridge M., Hopkins R.O., Angus D., Hart N., Hermans G., Iwashyna T., Arabi Y., Citerio G., Ely E.W. (2017). The ICM research agenda on intensive care unit-acquired weakness. Intensive Care Med..

[26] Miró O., Salmerón J.M., Masanés F., Alonso J.R., Graus F., Mas A., Grau J.M. (1999). Acute quadriplegic myopathy with myosin-deficient muscle fibres after liver transplantation: Defining the clinical picture and delimiting the risk factors. Transplantation.

[27] Wijdicks E.F., Litchy W.J., Wiesner R.H., Krom R.A. (1996). Neuromuscular complications associated with liver transplantation. Muscle Nerve.

[28] Rezaiguia-Delclaux S., Lefaucheur J.P., Zakkouri M., Duvoux C., Duvaldestin P., Stéphan F. (2002). Severe acute polyneuropathy complicating orthotopic liver allograft failure. Transplantation.

[29] Jang M.H., Yoon M.H., Ahn S.J., Lee J.W., Shin M.J. (2018). Diagnosis of Critical Illness Myopathy after Liver Transplantation and Muscle Condition Monitoring: A Case Report. Transplant. Proc..

[30] Watanabe J., Ito E., Hatano M., Tohyama T., Okada Y., Takada Y. (2016). Recovery after critical illness polyneuropathy in a patient with orthotopic liver transplantation: A case report. Transplant. Proc..

[31] Campellone J.V., Lacomis D., Giuliani M.J., Kramer D.J. (1998). Mononeuropathies associated with liver transplantation. Muscle Nerve.

[32] Kimura J. (2013). Electrodiagnosis in Diseases of Nerve and Muscle: Principles and Practice.

[33] Avolio A.W., Lai Q., Cillo U., Romagnoli R., De Simone P. (2021). L-GrAFT and EASE scores in liver transplantation: Need for reciprocal external validation and comparison with other scores. J. Hepatol..

[34] Avolio A.W., Agnes S., Chirico A.S., Castagneto M. (1999). Primary dysfunction after liver transplantation: Donor or recipient fault?. Transplant. Proc..

[35] Zaragoza-García I., Arias-Rivera S., Frade-Mera M.J., Martí J.D., Gallart E., San José-Arribas A., Velasco-Sanz T.R., Blazquez-Martínez E., Raurell-Torredà M. (2023). Enteral nutrition management in critically ill adult patients and its relationship with intensive care unit-acquired muscle weakness: A national cohort study. PLoS ONE.

[36] Hermans G., De Jonghe B., Bruyninckx F., Van den Berghe G. (2008). Clinical review: Critical illness polyneuropathy and myopathy. Crit. Care.

[37] Puthucheary Z.A., Phadke R., Rawal J., McPhail M.J., Sidhu P.S., Rowlerson A., Moxham J., Harridge S., Hart N., Montgomery H.E. (2015). Qualitative Ultrasound in Acute Critical Illness Muscle Wasting. Crit. Care Med..

[38] Avolio A.W., Gaspari R., Teofili L., Bianco G., Spinazzola G., Soave P.M., Paiano G., Francesconi A.G., Arcangeli A., Nicolotti N. (2019). Postoperative respiratory failure in liver transplantation: Risk factors and effect on prognosis. PLoS ONE.

[39] Avolio A.W., Agnes S., Cillo U., Lirosi M.C., Romagnoli R., Baccarani U., Zamboni F., Nicolini D., Donataccio M., Perrella A. (2012). http://www.D-MELD.com, the Italian survival calculator to optimize donor to recipient matching and to identify the unsustainable matches in liver transplantation. Transpl. Int..

[40] Annicchiarico B.E., Siciliano M., Avolio A.W., Caracciolo G., Gasbarrini A., Agnes S., Castagneto M. (2008). Treatment of chronic hepatitis C virus infection with pegylated interferon and ribavirin in cirrhotic patients awaiting liver transplantation. Transplant. Proc..

[41] Gaspari R., Teofili L., Aceto P., Valentini C.G., Punzo G., Sollazzi L., Agnes S., Avolio A.W. (2021). Thromboelastography does not reduce transfusion requirements in liver transplantation: A propensity score-matched study. J. Clin. Anesth..

[42] Alhamar M., Uzuni A., Mehrotra H., Elbashir J., Galusca D., Nagai S., Yoshida A., Abouljoud M.S., Otrock Z.K. (2023). Predictors of intraoperative massive transfusion in orthotopic liver transplantation. Transfusion.

[43] Lapisatepun W., Ma C., Lapisatepun W., Agopian V., Wray C., Xia V.W. (2023). Super-massive transfusion during liver transplantation. Transfusion.

[44] Massicotte L., Carrier F.M., Denault A.Y., Karakiewicz P., Hevesi Z., McCormack M., Thibeault L., Nozza A., Tian Z., Dagenais M. (2018). Development of a Predictive Model for Blood Transfusions and Bleeding During Liver Transplantation: An Observational Cohort Study. J. Cardiothorac. Vasc. Anesth..

[45] Moore H.B., LaRiviere W., Rodriguez I., Brown K., Hadley K., Pomposelli J.J., Adams M.A., Wachs M.E., Conzen K.D., Kennealey P.T. (2023). Early predictors of prolonged intensive care utilization following liver transplantation. Am. J. Surg..

[46] Moore H.B., Saben J., Rodriguez I., Bababekov Y.J., Pomposelli J.J., Yoeli D., Ferrell T., Adams M.A., Pshak T.J., Kaplan B. (2023). Postoperative fibrinolytic resistance is associated with early allograft dysfunction in liver transplantation: A prospective observational study. Liver Transpl..

[47] Duehrkop C., Rieben R. (2014). Refinement of tourniquet-induced peripheral ischemia/reperfusion injury in rats: Comparison of 2 h vs 24 h reperfusion. Lab. Anim..

[48] Goossens C., Weckx R., Derde S., Dufour T., Vander Perre S., Pauwels L., Thiessen S.E., Van Veldhoven P.P., Van den Berghe G., Langouche L. (2019). Adipose tissue protects against sepsis-induced muscle weakness in mice: From lipolysis to ketones. Crit. Care.

[49] Tandon P., Zanetto A., Piano S., Heimbach J.K., Dasarathy S. (2023). Liver transplantation in the patient with physical frailty. J. Hepatol..

[50] Hermans G., Clerckx B., Vanhullebusch T., Segers J., Vanpee G., Robbeets C., Casaer M.P., Wouters P., Gosselink R., Van Den Berghe G. (2012). Interobserver agreement of Medical Research Council sum-score and handgrip strength in the intensive care unit. Muscle Nerve.

[51] Vanpee G., Segers J., Van Mechelen H., Wouters P., Van den Berghe G., Hermans G., Gosselink R. (2011). The interobserver agreement of handheld dynamometry for muscle strength assessment in critically ill patients. Crit. Care Med..

[52] Hermans G., De Jonghe B., Bruyninckx F., Van den Berghe G. (2014). Interventions for preventing critical illness polyneuropathy and critical illness myopathy. Cochrane Database Syst. Rev..

[53] Hermans G., Van den Berghe G. (2015). Clinical review: Intensive care unit acquired weakness. Crit. Care.

